# The relation between health insurance and management of hypertension in Shanghai, China: a cross-sectional study

**DOI:** 10.1186/s12889-016-3627-3

**Published:** 2016-09-10

**Authors:** Xinji Zhang, Yuan Zhang, Xiang Xiao, Xiuqiang Ma, Jia He

**Affiliations:** Department of Health Statistics, Second Military Medical University, No. 800 Xiangyin Road, Shanghai, 200433 China

**Keywords:** Hypertension, Disease management, Health insurance

## Abstract

**Background:**

We aimed to investigate the management of hypertension in Shanghai, China and to examine whether there was any difference of hypertension management among people enrolled in different health insurances.

**Methods:**

In this cross-sectional study, a total of 31,531 residents were selected in Shanghai, using a randomized, stratified, multi-stage sampling method, and were asked to provide their status of hypertension, condition of hypertension management, health insurances and other demographic information. A weighted propensity score model was used to adjust confounders and to analyze the differences on hypertension management among hypertension patients enrolled in different health insurances.

**Results:**

In Shanghai, most hypertension patients achieved good management of hypertension. However, patients enrolled in the New Cooperative Medical Scheme or the Urban Resident Basic Medical Insurance scheme were more likely to achieve publicity of precautionary knowledge about hypertension (OR = 2.36 [95 % CI :1.96,2.85] and 1.28 [95 % CI:1.12,1.45], respectively) and had their blood pressure under control (OR = 1.33 [95 % CI :1.09,1.62] and 1.22 [95 % CI:1.05,1.42], respectively) than patients enrolled in the Urban Employee Basic Health Insurance scheme.

**Conclusion:**

The study provided a comprehensive description of hypertension in Shanghai, China. To support the management of hypertension, publicity of hypertension prevention knowledge should be improved, especially to people enrolled in the Urban Employee Basic Health Insurance scheme.

**Electronic supplementary material:**

The online version of this article (doi:10.1186/s12889-016-3627-3) contains supplementary material, which is available to authorized users.

## Background

Hypertension is one of the most important risk factors and the leading cause for cardiovascular disease (CVD) worldwide [1] and is also a growing burden to China’s public health. It was reported that the number of patients nationwide has increased from less than 100 million in 1991 to 260 million in 2012 [2]. The prevalence of hypertension among adults aged >18 year was 18.8 % according to a National Survey in 2002, and increased to approximately 25 % in recent years [3]. The greater the risk, the greater the benefit of hypertension control [4].

Effective management of hypertension is a core requirement of any health system [5] and previous studies demonstrated that the lacking in insurance coverage is a risk factor of management of hypertension [6, 7]. In China, with the reform of health insurance in recent years, almost residents already had health insurance [8]. Health insurance can improve hypertension management by increasing the number of people receiving all categories of prevention services without much cost and by lowering patients’ out-of-pocket costs of antihypertensive drugs [6]. However, in China, residents may achieve different types of health insurance based on their employment status, household registration systems and living locations [8]. In China, there are three main types of health insurance, the Urban Employee Basic Health Insurance scheme (UEBMI, launched in 1998), the New Cooperative Medical Scheme (NCMS; launched in 2003) and the Urban Resident Basic Medical Insurance scheme (URBMI; launched in 2007). The UEBMI provides coverage to the employees and retirees in the public sector. The NCMS is to improve access to health insurance for rural population. The URBMI is for urban residents without formal employment [9].

Different health insurances are separately governed by different authorities and provide different benefits packages [8]. UEBMI, URBMI and NCMS cover the expenditure of hypertension care, if patients visited the medical center and used hypertension drugs which are appointed by the government. However, until recently, few studies have examined whether there is any difference of hypertension management among people enrolled in different health insurances.

To investigate these issues, we analyzed data of 7974 residents with hypertension based on the fifth Health Service Survey of Shanghai, which was the extension of China’s National Health Service Survey (NHSS).

## Methods

### Data source

The data used for analysis was from the fifth Health Service Survey of Shanghai, which had been organized and completed by the Shanghai Municipal Commission Health and Family Planning in 2013 and was the extension of NHSS. The NHSS was approved by the National Commission Health and Family Planning China every fifth year since 1993 [10] Until now, the NHSS has been conducted for five times (1993, 1998, 2001, 2003, 2008 and 2013). The detailed sampling and quality assurance measures used the uniform standards throughout the nationwide scope and have been described in detail elsewhere [11] and are briefly summarized here. To achieve representation of the whole population in Shanghai, a three-stage, stratified, random sampling method was adopted. In the first stage, 100 towns/townships (sub-district) were selected randomly from all of the 17 counties/districts in Shanghai. The second stage sampled villages/communities. One thousand villages/communities were selected in the sampled towns/townships (sub-district). In the third stage, 12,000 households were identified. A face-to-face interview was conducted for each household, using a structured household questionnaire which was developed by the National Commission Health and Family Planning of China. The household questionnaire contained general information on socio-economic and demographic characteristics of the households; insurance characteristics of household members; self-reported illness and injury; outpatient and inpatient health service utilization.

Considerable quality assurance measures were implemented during the process of data collection. Survey constitutors checked the data to find out whether there were logic errors. If logic errors were found out, investigators would visit the corresponding household again to verify relevant information. Besides, survey constitutors revisited 5 % of the sampled households to check the accuracy of data recorded by interviewers. In this process, ten key questions were asked again to check the consistency of the information recorded. The consistency rates of the key questions recorded between the first and second visits was near 99 %. The Myer’s Blended index was 7.39, indicating that there was non-existence of age preference in this survey [12].

At last, we got the information of 31,531 respondents. However, we just focused on the adult and senior samples defined as those aged ≥ 18 years old (*n* = 28,672).

### Data collection

#### Hypertension related information

Every resident was asked to complete a comprehensive health and lifestyle questionnaire and indicated whether they had hypertension based on the question “Have you ever been told by a doctor or health professional that you have hypertension?” The management of hypertension on patients included measuring blood pressure within a month, publicity of hypertension prevention knowledge within 3 months, taking antihypertensive drugs every day and having blood pressure controlled. “Publicity of hypertension prevention knowledge” means regular hypertension health education. The aim of the health education is to raise awareness of three level prevention of hypertension, namely, primary prevention for health population, secondary prevention for high-risk population, and tertiary prevention for patients. The knowledges include information about improving self-care awareness, promoting a healthy lifestyle, and preventing and controlling the various risk factors of hypertension and so on [13]. The information of having blood pressure under control was collected from the answer to the question: “Is your blood pressure under controlled?”. Hypertension patients can choose one from three answer choices: “Yes”, “No” or “Not clear”. Other information of hypertension management was collected from the answer to the relevant questions.

All of the above information was self-reported.

#### Health insurance information

Participants were queried on their type (s) of health insurance coverage, which included UEMRI, URBMI, NCMS and other types of health insurance. The health insurance section contained several questions as following: “Do you participate in the UEBMI?”, “Do you participate in the URBMI”, “Do you participate in the NCMS”, “Do you participate in the cooperative medical insurance for urban and rural residents”, “Do you participate in any commercial insurances” and “Do you participate in any other kinds of health insurance”. Residents can participant only one of the three main health insurances (URBMI, UEBMI, NCMS). Respondents will be definded as uninsured if they did not participant any type of health insurance.

#### Information of outpatient health services

Every participant was questioned about the information of health condition in the past 2 weeks, including the presence or absence of diseases, whether sought outpatient health services, the expenditure of outpatient health services, the self-evaluation of the expenditure and so on.

#### Social-demographic information

Social-demographic characteristics considered in this study included sex, age, marital status, education attainment, residential areas and annual household income. In addition, we also considered physical activity (if respondents engaged in physical activities at least once a week in the last 6 months) and other morbidities (for example: whether respondents had been diagnosed with diabetes, musculoskeletal problems, respiratory disease or cancer).

### Statistical analyses

Descriptive statistics were summarized using mean (standard deviation, SD) for each numerical variables and counts (percentages) for categorical variables. Chi-square test was used to compare the difference of hypertension management among patients with different health insurances (measuring blood pressure within a month, publicity of hypertension prevention knowledge within 3 months, taking antihypertensive drugs every day and having blood pressure controlled). Outpatient services expenditures of hypertension patients with different health insurances were compared by using Kruskal-Wallis H test.

Different logistic regressions were used to calculate the odds ratios (ORs) for four outcomes: got measuring blood pressure within a month, achieved publicity of hypertension prevention knowledge within 3 months, took antihypertensive drugs every day and had blood pressure controlled, respectively. The patients with UEBMI were used as a reference group in the calculations of the ORs for other patients with different types of health insurance.

To adjusted confounders, an inverse probability of treatment weighting (IPTW) method was used [14]. The propensity score (PS), a probability of a respondent participating corresponding health insurance, was estimated by a multiple logistic regression model. The PS model contained several variables, including age, sex, education status, physical exercise status, annual household income, smoking status, drinking status, physical exercises status, household type and body mass index (BMI). This score was used in the multivariable analyses for the outcome using the IPTW method. When using the IPTW method, a stabilized weight was used to maintain the number of respondents used in the weighted analysis as the number of original study respondents [14]. A series of univariate regressions were performed to assess the likelihood of balancing baseline covariates before and after applying the IPTW method. The confounders which were not balanced after implementation of the propensity score were also included in the outcome model for more accurate estimation [15]. Besides, when estimating the ORs of having blood pressure controlled, we added status of measuring blood pressure within a month, achieved publicity of hypertension prevention knowledge and taking antihypertensive drugs every day into outcome model to adjust their effect on the outcome. Meanwhile, status of measuring blood pressure within a month, achieved publicity of hypertension prevention knowledge were included into the outcome model when we estimated the ORs of taking antihypertensive drugs every day. Furthermore, several logistic regressions for outcomes with different specifications were performed as sensitivity analyses. The results of all sensitivities analyses and balance analysis of confounders were presented in the appendix files (Additional file 1: Table S1 and Additional file 2: Table S2).

*P* values reported were two-tailed and a *p* value < 0.05 was considered statistically significant. All statistical analyses were performed using SAS software (version 9.4; SAS Institute Inc., Cary, NC).

## Result

### Health insurance sources

Of all 28,672 residents aged ≥18 years old, 27.2 % (*n* = 7974) had hypertension. A total of 98.9 % of these patients (*n*﻿ = 7883) participate in health insurances. The detail information of health insurance sources in different subgroups was shown in Table 1. Among the patients with hypertension, 53.8 % (*n* = 4293) were enrolled in UEBMI, 27.5 % (*n* = 2192) were enrolled in URBMI and 16.0 % (*n* = 1276) were enrolled in NCMS. 3 hypertension patients with insurance missed household type information (0.04 %) and 12 residents with hypertension missed physical exercise information (0.15 %).

**Table 1:** Different health insurances in residents ≥18 years with hypertension by demography

	Number (%)				
	All^a^	UEBMI	URBMI	NCMS	Others^b^
	*N* = 7883	*N* = 4293	*N* = 2192	*N* = 1276	*N* = 122
Sex					
Male	3855 (48.9)	2324 (54.1)	950 (43.3)	530 (41.5)	51 (41.8)
Female	4028 (51.1)	1969 (45.9)	1242 (56.7)	746 (58.5)	71 (58.2)
Household type^c^					
Agricultural	1577 (20.0)	222 (5.2)	162 (7.4)	1169 (91.6)	24 (19.7)
Non-agricultural	6304 (80.0)	4069 (94.8)	2030 (92.6)	107 (8.4)	98 (80.3)
Age					
18–29	13 (0.2)	10 (0.2)	2 (0.1)	1 (0.1)	0 (0.0)
30–39	77 (1.0)	64 (1.5)	5 (0.2)	8 (0.6)	0 (0.0)
40–49	450 (5.7)	303 (7.1)	89 (4.1)	48 (3.8)	10 (8.2)
50–64	3308 (42.0)	1853 (43.2)	894 (40.8)	516 (40.4)	45 (36.9)
≥65	4035 (51.2)	2063 (48.1)	1202 (54.8)	703 (55.1)	67 (54.9)
Education					
Less than high school	5303 (67.3)	2265 (52.8)	1722 (78.6)	1236 (96.9)	80 (65.6)
High school graduate	2153 (27.3)	1674 (39)	404 (18.4)	40 (3.1)	35 (28.7)
higher than high school	427 (5.4)	354 (8.3)	66 (3.0)	0 (0.0)	7 (5.7)
Family income (year)					
≤48,000	2716 (34.5)	865 (20.2)	912 (41.6)	901 (70.6)	38 (31.2)
48,001 ~ 70,000	2186 (27.7)	1398 (32.6)	573 (26.1)	188 (14.7)	27 (22.1)
70,001 ~ 100,000	1684 (21.4)	1119 (26.1)	416 (19.0)	123 (9.6)	26 (21.3)
>100,000	1297 (16.5)	911 (21.2)	291 (13.3)	64 (5.0)	31 (25.4)
Physical exercises status^d^					
more than 6 times a week	2259 (28.7)	1448 (33.8)	621 (28.4)	151 (11.9)	2259 (28.7)
3–5 times a week	997 (12.7)	607 (14.2)	263 (12.0)	115 (9.0)	997 (12.7)
1–2 times a week	688 (8.7)	398 (9.3)	191 (8.7)	88 (6.9)	688 (8.7)
Less than 1 time a week	241 (3.1)	149 (3.5)	57 (2.6)	29 (2.3)	241 (3.1)
Never	3686 (46.8)	1686 (39.3)	1055 (48.2)	891 (69.9)	3686 (46.8)
Diabetes					
Yes	1306 (16.6)	779 (18.1)	337 (15.4)	167 (13.1)	23 (18.9)
No	6577 (83.4)	3514 (81.9)	1855 (84.6)	1109 (86.9)	99 (81.1)

Note:

*UEBMI* the urban employee basic health insurance scheme, *URBMI* the urban resident basic medical insurance, *NCMS* the new cooperative medical scheme

The unit of family income is China Yuan (CNY)

^a^ all hypertension patients with any types of insurances

^b^ Other health insurance means residents didn’t use UEBMI, URBMI or NCMS, but just use other types of health insurance;

^c^ three hypertension patients with insurance missed household type information

^d^ 12 hypertension patients with insurance missed physical exercises status information

### Implementation of hypertension management

Among the residents with hypertension, 85.8 % (*n* = 6845) had their blood pressure under control. Most of residents with hypertension took antihypertensive drugs every day (*n* = 7216, 90.5 %) and measured their blood pressure within a month (*n* = 7138, 89.5 %).78.8 % of the residents with hypertension (*n* = 6284) achieved publicity of hypertension prevention knowledge within 3 months.

The differences in the management of hypertension among patients with different health insurances were shown in Table 2. Patients enrolled in UEBMI had the highest proportion of having measured blood pressure measurement within a month (90.5 %), following by patients enrolled in URBMI (89.6 %) and patients enrolled in NCMS had the lowest proportion (86.7 %). The difference on the percentage of getting blood pressure measurement within a month among patients enrolled in different health insurances was statistically significant (*p* < 0.001). There was also statistically significant difference on the proportion of achieving publicity of hypertension prevention knowledge among hypertension patients enrolled in different health insurances (74.9 % for UEBMI, 81.1 % for URBMI and 88.4 % for NCMS, *p* < 0.001). The percentages of patients taking antihypertensive drugs every day were similar among patients with different  health insurances (89.9 % in patients with UEBMI, 91.2 % in patients with URBMI and 91.5 % in patients with NCMS, *p* = 0.109). However, there was significant statistical difference on the proportion of controlled blood pressure (85.7 % in patients with UEBMI, 87.1 % in patients with URBMI and 84.1 % in patients with NCMS, *p* = 0.041).

**Table 2:** Hypertension management in patients with different insurances

	Blood pressure measurement^a^	Prevention knowledge^b^	Antihypertensive drugs^c^	Blood pressure under control^d^
All (*n* = 7883)	7064 (89.6 %)	6213 (78.8 %)	7131 (90.5 %)	6765 (85.8 %)
UEBMI (*n* = 4293)	3884 (90.5 %)	3216 (74.9 %)	3858 (89.9 %)	3678 (85.7 %)
URBMI (*n* = 2192)	1965 (89.6 %)	1778 (81.1 %)	1998 (91.2 %)	1909 (87.1 %)
NCMS (*n* = 1276)	1107 (86.7 %)	1128 (88.4 %)	1167 (91.5 %)	1072 (84.1 %)
Others (*n* = 122)	108 (88.5 %)	91 (74.6 %)	108 (88.5 %)	105 (86.1 %)

Note:

*UEBMI* the urban employee basic health insurance scheme, *URBMI* the urban resident basic medical insurance, *NCMS* the new cooperative medical scheme

^a^ patients measured their blood pressure within a month;

^b^ patients achieved publicity of hypertension prevention knowledge;

^c^: patients took antihypertensive drugs every day;

^d^ patient had their blood pressure under control

In regression analysis, of the 7883 records of hypertensive insured respondents, 18 have missing data and the missingness rate is 0.23 %. After adjusted for confounders, compared with hypertension patients with UEBMI, hypertension patients with URBMI were more likely to achieve publicity of hypertension prevention knowledge within 3 months (OR:1.28, 95 % Cl:1.12–1.45) and had their blood pressure under control (OR:1.22,95 % Cl:1.05,1.42). Meanwhile, there were similar phenomena among patients with NCMS. Patients with NCMS were also more likely to achieve publicity of precautionary knowledge about hypertension within 3 months (OR: 2.36, 95 % Cl: 1.96, 2.85) and had their blood pressure under control (OR: 1.33, 95 % Cl: 1.09, 1.62). There were no difference on the control of blood pressure between patients with NCMS and URBMI (Table 3).

**Table 3:** Regression Analyses of the implementation of management of hypertension patients

	Blood pressure measurement^a^		Publicity of hypertension prevention knowledge^b^		Antihypertensive drugs^c^		Blood pressure under control ^d^	
	Adjusted OR (95 % CI)	*p* value	Adjusted OR (95 % CI)	*p* value	Adjusted OR (95 % CI)	*p* value	Adjusted OR (95 % CI)	*p* value
Health insurance (vs UEBMI)								
URBMI	0.99 (0.84,1.18)	0.924	1.28 (1.12,1.45)	<0.001	0.94 (0.79,1.13)	0.510	1.22 (1.05,1.42)	0.015
NCMS	0.66 (0.54,0.80)	<0.001	2.36 (1.96,2.85)	<0.001	1.28 (1.00,1.65)	0.052	1.33 (1.09,1.62)	0.008
Others	0.86 (0.49,1.51)	0.595	0.93 (0.61,1.40)	0.715	0.79 (0.44,1.39)	0.407	1.21 (0.71,2.07)	0.558
Variables weren’t balanced after implementation of the PS								
Age	1.01 (1.01,1.02)	<0.001	1.01 (1.01,1.02)	<0.001	1.01 (1.01,1.02)	<0.001	1.00 (0.97,1.01)	0.411
BMI	1.02 (1.00,1.04)	0.073	1.01 (0.99,1.03)	0.239	1.07 (1.04,1.10)	<0.001	1.00 (0.98,1.02)	0.988
Sex (Male vs Female)	0.97 (0.81,1.17)	0.756	0.68 (0.58,0.80)	<0.001	1.10 (0.90,1.35)	0.366	1.15 (0.97,1.36)	0.121
Household type (Agricultural vs Non-agricultural)	1.03 (0.87,1.22)	0.7561	1.08 (0.95,1.23)	0.220	1.14 (0.95,1.37)	0.161	0.79 (0.68,0.92)	0.032
Drink (vs not drink)								
less than 1 time in a week	0.72 (0.57,0.90)	0.004	1.03 (0.86,1.24)	0.752	0.65 (0.51,0.83)	<0.001	0.68 (0.55,0.84)	<0.001
1–2 times in a week	0.73 (0.50,1.09)	0.123	1.16 (0.84,1.59)	0.373	0.65 (0.44,0.96)	0.029	0.61 (0.43,0.87)	0.006
More than 2 times a week	0.64 (0.45,0.89)	0.009	0.83 (0.64,1.08)	0.161	0.59 (0.42,0.83)	0.002	0.92 (0.65,1.29)	0.612
Physical exercises status (vs more than 6 times a week)								
3–5 times a week	0.87 (0.67,1.12)	0.267	0.88 (0.73,1.06)	0.183	0.71 (0.54,0.92)	0.010	0.75 (0.60,0.94)	0.011
1–2 times a week	1.24 (0.90,1.72)	0.188	0.86 (0.7,1.07)	0.170	0.54 (0.41,0.72)	<0.001	1.06 (0.81,1.40)	0.669
Less than 1 time a week	0.69 (0.47,1.02)	0.061	0.6 (0.45,0.79)	<0.001	0.93 (0.59,1.46)	0.749	1.14 (0.76,1.71)	0.538
Never	0.63 (0.53,0.75)	<0.001	0.91 (0.8,1.05)	0.190	0.76 (0.62,0.93)	0.009	0.73 (0.62,0.86)	0.001
Education (vs less than high school)								
high school graduate	1.81 (1.49,2.20)	<0.001	1.04 (0.91,1.18)	0.565	0.87 (0.72,1.03)	0.154	1.54 (1.29,1.82)	<0.001
higher than high school	1.24 (0.85,1.81)	0.270	0.98 (0.76,1.27)	0.882	0.75 (0.52,1.06)	0.104	1.07 (0.77,1.74)	0.700

Note:

*UEBMI* the urban employee basic health insurance scheme, *URBMI* the urban resident basic medical insurance, *NCMS* the new cooperative medical scheme, *PS* propensity score

These confounders in the outcome model are those variables are not balanced after implementation of the propensity score

^a^ patients measured their blood pressure within a month;

^b^ patients achieved publicity of precautionary knowledge on hypertension within three months;

^c^ patients took antihypertensive drugs every day;

^d^ patient had their blood pressure under control

### Expenditure of outpatient services due to hypertension

36.57 % of hypertension patients (*n* = 2916) turned to outpatient services for help due to hypertension within 2 weeks. The status of outpatient service expenditure among patients with three main health insurances was show in Table 4. There was no significant statistical difference on expenditure among three main health insurances, while the self-evaluation of the expenditures among the three groups showed significant statistical difference (*p* < 0.001). Higher proportion of patients with NCMS considered the cost of outpatient service cheap (58.89 % vs 33.80 % in UEBMI and 35.67 % in URBMI).

**Table 4:** Outpatient services expenditures of hypertension patients

		UEBMI	URBMI	NCMS
		*n* = 1631	*n* = 785	*n* = 434
Expenditure (median, IQR)		17 (5,35)	15 (0,40)	10 (5,30)
Evaluation of the expenditure	Cheap (n, %)	551 (33.80 %)	280 (35.67 %)	255 (58.89 %)
	Appropriate (n,%)	857 (52.58 %)	422 (53.76 %)	148 (34.18 %)
	Expensive (n, %)	222 (13.62 %)	83 (10.57 %)	30 (6.93 %)

Note:

*UEBMI* the urban employee basic health insurance scheme, *URBMI* the urban resident basic medical insurance, *NCMS* the new cooperative medical scheme

The unit of expenditure is China Yuan (CNY)

One patient with UEBMI and one patient with NCMS did not provides their evaluation about the expenditures

## Discussion

Our Study enriches the general literature of social health insurance [8, 9]. Using data collected in 2013 from the fifth Health Service Survey of Shanghai, we found that hypertension was a big burden to the local health system, but most hypertension patients achieved good management of hypertension and had their blood pressure under controlled. However, our results demonstrated that there were some differences on the management of hypertension among patients with different health insurances.

Our result showed that 27.2 % of residents who were aged ≥18 years old had hypertension, lower than the national level (33.6 %) [16]. Because of the self-reported hypertension status, the actual prevalence of hypertension in Shanghai would be higher than the level in our study. Among the hypertension patients, most of them measured blood pressure within a month (89.5 %), took antihypertensive drugs every day (90.5 %) and had their blood pressure under control (85.8 %). The blood pressure controlled rate was much higher than the reported nation level (approximately 20 % patients with blood pressure under control) [16, 17]. Self-report could overestimate the blood pressure controlled rate, and another possible explanation is that the blood pressure controlled rate in our study is estimated based on the respondents with hypertension awareness. Of those individuals with hypertension in the national survey, only one quarter interviewees were aware of their condition let alone taking measure to control hypertension. Actually, our results were similar to the results of Li’s previous study [18] based on registered hypertensive patients in Shanghai (measured blood pressure controlled rate: 83.2 %). The rate of drug treatment (90.5 %) is also similar to that of their study (95.2 %). Higher health insurance coverage is associated with improved drug treatment rate and medication adherence which may translate into better control of hypertension.

An interesting finding of our study was that patients enrolled in URBMI or NCMS were more likely to have their blood pressure under control as compared with those enrolled in UEBMI in Shanghai. Even though patients enrolled in different insurances had similar proportion of taking antihypertensive drugs every day. These results were different from those of previous studies which suggested that patients with UEBMI provided better benefit package and had better control of non-communicable diseases (NCDs) than those with URBMI and NCMS [9, 17]. Due to the complicacy of hypertension management, the difference of blood pressure control among different insurances can be attributed to many potential causes. More of the patients enrolled in UEBMI were male (54.13 % vs 43.34 % in URBMI and 41.54 % in NCMS). In some studies [19, 20], male had lower proportion of effective hypertension management than female, which might reduce the efficiency of hypertension management in patients enrolled in UEBMI. Another potential reason is the big differences in precautionary knowledge on hypertension. Our results demonstrated that patients enrolled in URBMI or NCMS were more likely to achieved publicity of precautionary knowledge on hypertension than patients enrolled in UEBMI. However, after adjusted for demographic confounders and the status of measuring blood pressure within a month, achieved publicity of hypertension prevention knowledge and taking antihypertensive drugs every day, the differences still remained. Another possible explanation might be that unlike URBMI and NCMS, UEBMI had covered the expenditures in pharmacy. Patients enrolled in UEBMI can access antihypertensive drugs more conveniently from pharmacy, while patients enrolled in URBMI or NCMS have to go to community health service centers or general hospitals for their antihypertensive drugs. Thus, patients with UEBMI insurance might get less professional medical advice on individual-based management of hypertension if they got antihypertensive drugs from pharmacy. Previous study also suggested that having a regular place to receive medical care was an important factor for hypertension control [13]. Self-management is an indispensable part in hypertension management. To improve the level of hypertension management, adequate knowledge on hypertension and effective self-management are as important as effective drug therapy [13, 21].

In the aspect of outpatient services expenditure due to hypertension, there was similar outpatient service expenditure among patients enrolled in different insurances. Meanwhile, more patients enrolled in NCMS considered the expenditure cheap than patients enrolled in UEBMI or URBMI. However, in China, reimbursement ratio was usually lower for NCMS than for either URBMI or UEBMI [22]. There are two reasons which may account for this phenomenon. First, hypertension drugs are usually cheap and most of the expenditure can be reimbursed. However, NCMS patients may get fewer subsidies on other more expensive drugs or therapies. By comparing the actual expenditure on hypertension drugs to other drugs or therapies, they may feel the expenditure of hypertension drugs cheaper. Second, NCMS patients have much lower annual household income and the ratio of subsidy for hypertension drugs to annual household income in NCMS patients is higher than that of UEBMI or URBMI patients, so NCMS patients are more likely to consider the expenditure “cheap”.

There are some inevitable limitations in this study. First, most information is self-reported which could lead to an underestimation of the prevalence of hypertension. Previous researchers [23] have compared the agreement between biomedical and self-reported measurements of hypertension of a Chinese national community sample. And they suggested self-reported data can be a reliable estimate of the prevalence of hypertension in developed communities or provinces. Nevertheless, caution should be taken when applying our results Second, we did not take patients only had other types of insurance into consideration because most patients (97.3 %) were enrolled in NCMS, URBMI or UEBMI. Third, while propensity score weight is useful for controlling for observed covariates, it is not helpful to control for unobserved covariates, except to the extent that they are correlated with the observed covariates. And due to the inherent methodological limitation of cross-section study, longitudinal quasi-experimental studies are needed to further confirm the differences of hypertension-related outcomes among the three types of health insurances.

## Conclusions

The study is a large-scale, population based study to investigate the differences of hypertension management among people enrolled in different health insurances in Shanghai, China. This cross-sectional study demonstrated that, in Shanghai, most hypertension patients achieved good management of hypertension. There might be some differences of hypertension management among people with different health insurances. Patients enrolled in URBMI or NCMS were more likely to achieve publicity of precautionary knowledge about hypertension within 3 months and were related to better management of hypertension than patients enrolled in UEBMI. Precautionary knowledge about hypertension should be disseminated widely, especially to people enrolled in UEBMI. Besides, there is still a lot of work to do in order to consolidate existing social health insurance schemes and achieve universal health coverage in China.

## Additional files

Additional file 1: Table S1. ORs in different outcome models. (DOCX 19 kb) Additional file 2: Table S2.
*P*-Values for the Differences Before and After Correction on the Multiple propensity score. (DOCX 15 kb)

## Abbreviations

BMI: Body mass index
CI: Confidence interval
CVD: Cardiovascular disease
IPTW: Inverse probability of treatment weighting
NCD: Non-communicable diseases
NCMS: The new cooperative medical scheme
OR: Odds ratios
PS: Propensity score
UEBMI: The urban employee basic health insurance scheme
URBMI: The urban resident basic medical insurance scheme
