# Supplementary material for: The relation between health insurance and management of hypertension in Shanghai, China: a cross-sectional study
Source: BMC Public Health. 2016 Sep 10;16(1):959. doi: 10.1186/s12889-016-3627-3 (PMC5018164; doi:10.1186/s12889-016-3627-3)
Supplement: Additional file 1: Table S1. — ORs in different outcome models. (DOCX 19 kb) [file 12889_2016_3627_MOESM1_ESM.docx]

**Table S1** ORs in different outcome models

| Outcome | Adjusted OR (95% CI) | | |
| --- | --- | --- | --- |
|  | URBMI vs UEBMI | NCMS vs UEBMI | Others vs UEBMI |
| Blood pressure measurement within a month ^a^ |  |  |  |
| Model 1 | 1.02(0.86,1.20) | 0.67(0.56,0.81) | 0.86(0.49,1.50) |
| Model 2 | - | - | - |
| Model 3 | 0.99(0.84,1.18) | 0.66(0.54,0.80) | 0.86(0.49,1.51) |
| Model 4 | 1.01(0.84,1.21) | 0.88(0.64,1.22) | 0.80(0.45,1.42) |
| Publicity of hypertension prevention knowledge ^a^ |  |  |  |
| Model 1 | 1.30(1.15,1.48) | 2.36(1.96,2.83) | 0.93(0.62,1.41) |
| Model 2 | - | - | - |
| Model 3 | 1.28(1.12,1.45) | 2.36(1.96,2.85) | 0.93(0.61,1.40) |
| Model 4 | 1.39(1.21,1.59) | 1.86(1.41,2.47) | 0.89(0.59,1.36) |
| Taking antihypertensive drugs ^b^ |  |  |  |
| Model 1 | 1.02(0.85,1.21) | 1.48(1.16,1.87) | 0.80(0.46,1.39) |
| Model 2 | 0.97(0.81,1.16) | 1.40(1.10,1.79) | 0.82(0.46,1.43) |
| Model 3 | 0.94(0.79,1.13) | 1.28(1.00,1.65) | 0.79(0.44,1.39) |
| Model 4 | 1.02(0.84,1.24) | 1.15(0.81,1.64) | 0.81(0.45,1.46) |
| Blood pressure under control ^c^ |  |  |  |
| Model 1 | 1.25(1.08,1.46) | 1.40(1.16,1.70) | 1.16(0.69,1.95) |
| Model 2 | 1.21(1.04,1.41) | 1.30(1.07,1.59) | 1.20(0.71,2.03) |
| Model 3 | 1.22(1.05,1.42) | 1.33(1.09,1.62) | 1.21(0.71,2.07) |
| Model 4 | 1.21(1.03,1.43) | 1.15(0.86,1.53) | 1.11(0.65,1.89) |

**Note :**

**Model 1:** the model of outcome only includes insurance and propensity score

**Model 2:** the model of outcomes includes insurance, propensity score and "upstream" outcome variables.

**Model 3:** the model of outcomes includes insurance, "upstream" outcome variables (monthly BP measurement, knowledge) and some sociodemographic covariates related to the outcomes which are not balanced after implementation of the propensity score.

**Model 4:** traditional logistic regression model includes insurance, "upstream" outcome variables and other sociodemographic covariates related to the outcomes

**a:** Model 2 and model 3 did not contain “upstream” outcome variables. Model 1 is same as Model 2.

**b:** The “upstream” outcome variables contain blood pressure measurement and publicity of hypertension prevention knowledge.

**c:** The “upstream” outcome variables contain blood pressure measurement ,taking antihypertensive drugs and publicity of hypertension prevention knowledge.
