# Supplementary material for: The relation between health insurance and management of hypertension in Shanghai, China: a cross-sectional study
Source: BMC Public Health. 2016 Sep 10;16(1):959. doi: 10.1186/s12889-016-3627-3 (PMC5018164; doi:10.1186/s12889-016-3627-3)
Supplement: Additional file 2: Table S2. — P-Values for the Differences Before and After Correction on the Multiple propensity score. (DOCX 15 kb) [file 12889_2016_3627_MOESM2_ESM.docx]

**Table 2** *P*-Values for the Differences Before and After Correction on the Multiple PS

| Variable | Type of Variables | Before Multiple PS Correction | After Multiple PS Correction |
| --- | --- | --- | --- |
| Age | Continuous Variables | <0.001 | <0.001 |
| BMI | Continuous Variables | <0.001 | 0.018 |
| Sex | Categorical Variables | <0.001 | <0.001 |
| Household type | Categorical Variables | <0.001 | 0.036 |
| Education status | Categorical Variables | <0.001 | <0.001 |
| Annual household income | Categorical Variables | <0.001 | 0.803 |
| Smoking status | Categorical Variables | <0.001 | 0.255 |
| Drinking status | Categorical Variables | <0.001 | <0.001 |
| Physical exercises status | Categorical Variables | <0.001 | 0.007 |
